# A giant cervical ganglioneuroma in a middle-aged man: a case report

**DOI:** 10.3389/fonc.2026.1609468

**Published:** 2026-03-26

**Authors:** Ce Ding, Siyu Liu, Guosen Sun, Yu Li, Hong Ding, Wei Zhao

**Affiliations:** 1 Baoding First Central Hospital, Baoding, HeBei, China; 2 Chengde Medical University, Chengde, HeBei, China; 3 Shandong Second Medical University, Weifang, Shandong, China; 4 Qingdao Binhai University, Qingdao, Shandong, China

**Keywords:** cervical masses, ganglion cells neuroma, neck, neurogenic tumors, tumor

## Abstract

Cervical ganglioneuroma is a relatively rare benign neurogenic tumor for which standardized evidence-based guideline is lacking. It is more common in women, adolescents, and children and presents as an asymptomatic neck mass. Preoperative imaging and needle biopsy are insufficient to confirm the diagnosis, making postoperative histopathological examination of a specimen the gold standard. Herein, we present the case of a middle-aged man with a giant ganglioneuroma of the neck, discussing the relevant diagnostic and therapeutic characteristics. The patient was a 43-year-old man who had been diagnosed with a left neck mass 6 years prior, without obvious symptoms, received no treatment. One year prior, the patient presented with aggravated speech with content sounds and was admitted at our hospital for relevant examinations and surgical treatment. The patient underwent transcervical tumor resection under general anesthesia. Symptoms such as Horner syndrome, left deviation of tongue protrusion, and dysphagia occurred after surgery. At follow-up 2 months after surgery, symptoms such as left deviation of tongue extension and dysphagia disappeared, and symptoms such as ptosis were alleviated to a certain extent. Surgical resection remains the primary treatment for cervical ganglioneuromas. For large tumors, the transcervical approach can not only completely resect the tumor but also contribute to postoperative recovery.

## 1 Introduction

Ganglioneuroma (GN) is a relatively rare sympathetic nervous system tumor that can affect a wide range of areas (1). GN is more common in women, and rarely occurs in the neck (2). Most patients present with asymptomatic masses (3). Histopathological examination of postoperative specimens remains the gold standard for diagnosis. Surgical resection is often performed as treatment modality, and postoperative recovery is good.

## 2 Case report

**Figure 1:**
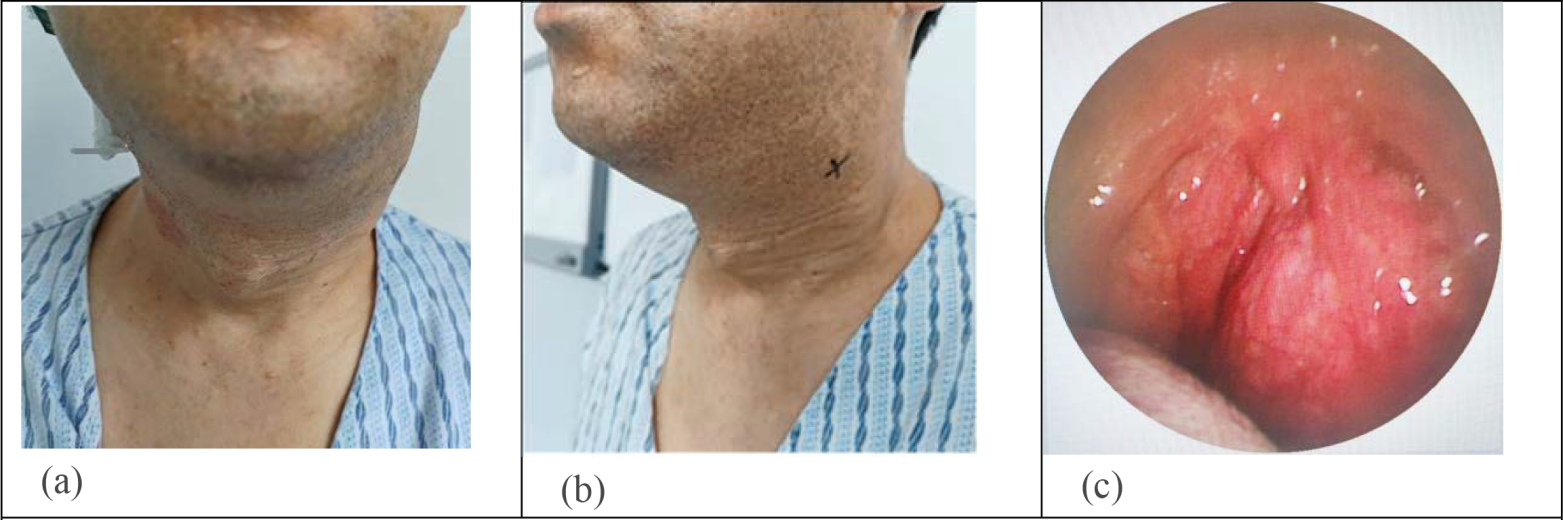
Images of the left neck mass. **(a, b)** A marked uplift of the left side of the neck could be seen. **(c)** The left lateral pharyngeal wall was clearly distended, crossing the midline, and the pharyngeal cavity was compressed and narrowed.

A 43-year-old male patient was admitted to the hospital with a history of a left neck mass for six years and dysarthria for one year. Six years prior, the patient incidentally noted a mass on the left neck, approximately the size of a red date, with slight material sounds in speech, occasionally accompanied by choking, without local swelling, pain, ulceration, limitation of mouth opening, dysphagia, dyspnea, cough, headache, or ptosis. During this period, the mass gradually increased. One year prior to admission, dysarthria worsened, accompanied by nasal sounds, without special treatment. Physical examination on admission revealed a narrow pharyngeal cavity, normal right uvula, no congestion in the right tonsil, grade II enlargement, bulging of the left lateral pharyngeal wall across the midline to the opposite side, a smooth mucosal surface. An epiglottis and laryngopharynx were unremarkable. Vascular pulsation was palpable on the left side of the neck. The thyroid cartilage deviated to the right, the left thyroid cartilage lamina was pushed up by the mass, and the trachea also deviated to the right. Jugular vein distension or abnormal carotid artery pulsation was not observed (**Figure 1**).

**Figure 2:**
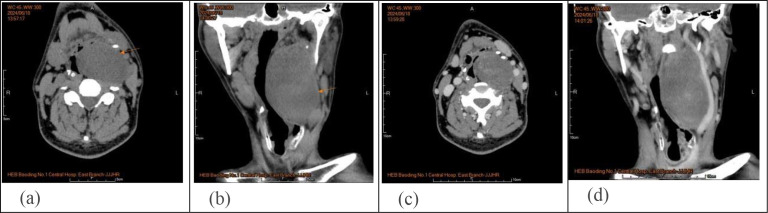
**(a,b)** CT of the neck: On the left side, an ovular, slightly low-density shadow, about 9.3*5.7*4.1cm in size, with a clear boundary, above the soft palate to the level of pyriform fossa, CT value of 15-39HU. Uneven density was observed between parapharyngeal and jugular vascular sheath. **(c, d)** Enhanced CT of the neck: The left common carotid artery, internal carotid artery and external carotid artery were compressed, and the left jugular vein was not clearly displayed.

**Figure 3:**
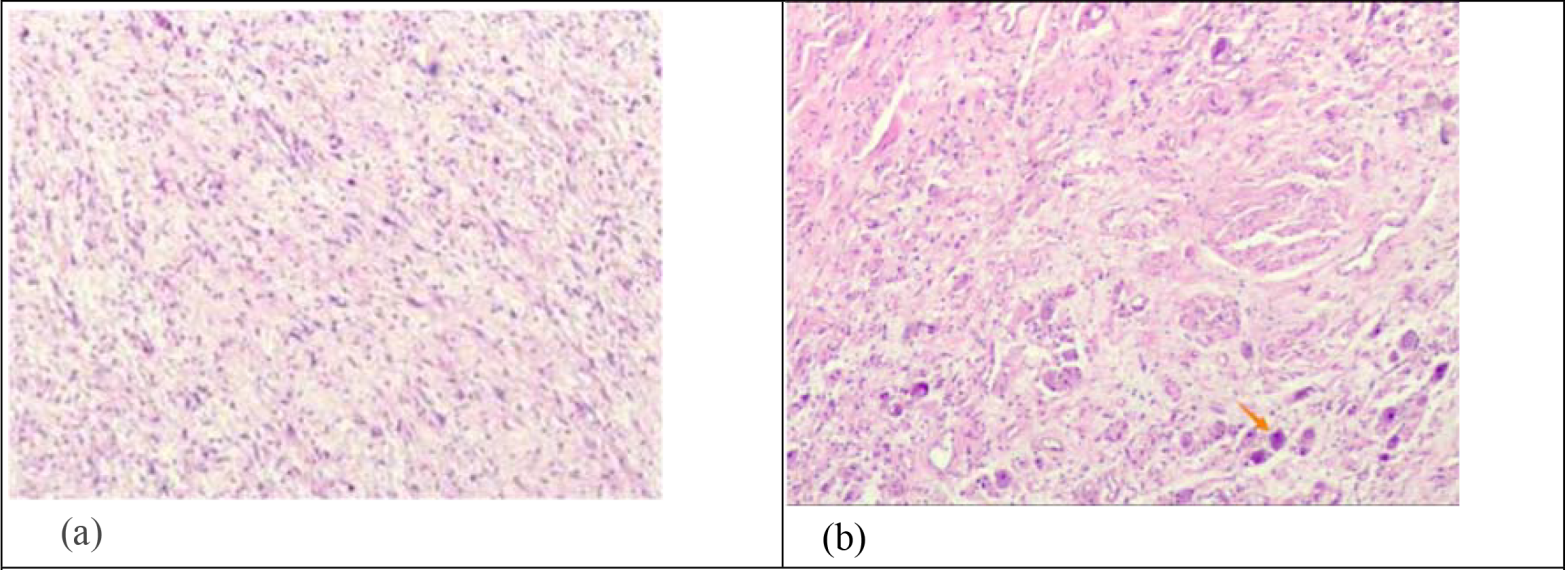
**(a)** Preoperative needle aspiration cytology showing spindle cell tumor cells, combined with immunohistochemistry, with results indicating schwannoma or neurofibroma. **(b)** Histopathological examination of postoperative specimens; when combined with morphology and immunohistochemistry, the results were consistent with ganglioneurofibroma. The arrow indicates ganglion cell.

Contrast-enhanced CT of the neck showed an oval, slightly low-density shadow between the left parapharyngeal and carotid vascular sheath, approximately 9.3*5.7*4.1 cm in size (**Figure 2**). The surrounding tissue was compressed; the left common carotid artery and the internal and external carotid arteries were compressed outward; and the left jugular vein was partially obscured. MRI of the neck revealed a mass shadow with long T1 and T2 signals in the left parapharyngeal space, for which the signal was heterogeneous. Multiple patches and other T2 signal shadows could be seen within the mass, and the enhanced scan showed marked heterogeneous enhancement. Ultrasound-guided puncture biopsy reveals spindle cell tumor cells. Due to the small amount of punctured tissue, a definitive diagnosis cannot be established. A preliminary diagnosis of schwannoma or neurofibroma is considered. No surgical contraindications were observed before surgery. The tumor was resected through the neck under general anesthesia, with the resected specimen subsequently sent to pathology. Pathological diagnosis: Ganglioneuroma (**Figure 3**). Immunohistochemical staining showed CD34 (partial +), S-100 (+), SOX10 (+), STAT6 (-), Ki-67 (1%), Pgp9.5 (+), SMA (-), Desmin (-), EMA (-). Postoperatively, the patient presented with clinical manifestations including upper eyelid ptosis, miosis, and facial anhidrosis, accompanied by tongue deviation to the left and dysphagia. At the 2-month follow-up, the tongue deviation and dysphagia had completely resolved, and the ptosis and other related symptoms were markedly relieved. The patient reported no hoarseness or other discomfort, and neurological examination revealed no abnormalities of other cranial nerves. Evaluation of Horner’s syndrome showed significant improvement in the associated signs compared with the early postoperative period. The patient recovered well after surgery, with no postoperative recurrence as of February 2025 (7 months postoperatively).

## 3 Discussion

GN is a relatively rare benign neurogenic tumor that originates from primitive neural crest cells, and is mainly composed of well-differentiated Schwann cells, ganglion cells, nerve fibers, and mucinous matrix (1). The malignancy rate is less than 1%, with a slight elevation only noted in cases involving large tumors, pathological presence of immature components, or concurrent genetic syndromes. Once malignant transformation occurs, the lesion may progress to a malignant peripheral nerve sheath tumor (4), which is typically characterized by rapid enlargement over a short period, accompanied by persistent pain, progressive neurological deficits, and systemic cachexia. Notably, these clinical manifestations often indicate aggressive disease progression and require timely clinical intervention. Existing studies on GN are primarily based on records and case reports, the reviews by Yang T and Xu T summarized 27 and 58 cases, respectively (3, 5). GN usually occurs in the abdomen and pelvis, but can also rarely present in the neck, and is most common in adolescents and children (2). Case reports of giant cervical GN in middle-aged men are rare. Most patients present with asymptomatic masses, and a few present with compression of the surrounding tissues, blood vessels, and nerves, accompanied by persistent dyspnea, dysphagia, cough, and Horner syndrome. There have also been reports indicating that some functional ganglioneuromas may secrete catecholamines, leading to clinical manifestations including flushing, hypertension, and diarrhea (3). In our case, the patient presented with an asymptomatic mass.

Histopathological examination of postoperative specimens is the gold standard for the diagnosis of GN, with diagnosis made if ganglion cells are identified in the tumor tissue (**Figure 3**). However, it is easily misdiagnosed as a spindle cell tumor because of the small number of ganglion cells and inadequate pathological evaluation. Fine-needle aspiration cytology (FNAC), as the first-line examination for cervical tumors, can help to identify the nature of the tumor and provide direction for the formulation of further surgical plans; however, it is still not definitive (**Figure 3**). Other researchers believe that severe pain during puncture is an important clue for the diagnosis of ganglioneuromas (6). In our patient, fine−needle aspiration biopsy showed spindle cells without significant atypia. Due to the limited amount of punctured tissue, the lesion was considered to represent schwannoma or neurofibroma preoperatively. Postoperative histopathological examination of the specimen, combined with morphology features and immunohistochemistry findings, showed a small number of ganglion cells, consistent with GN.

**Figure 4:**
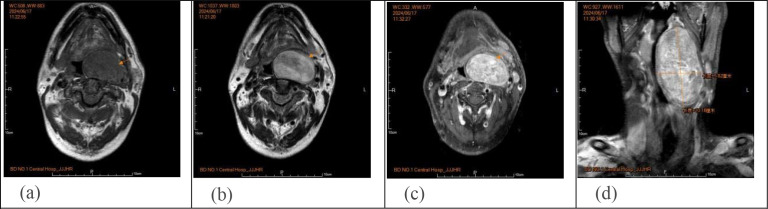
**(a, b)** MRI of the neck: Long T1 and T2 signal mass shadows can be seen in the left parapharyngeal space, with uneven signals, and T2 signal shadows, such as multiple plaques were seen in the space. The lesion was about 5.7×3.9×10.0cm in size, and the boundary was clear and smooth. **(c, d)** Enhanced MRI of the neck: Contrast-enhanced scan showed obvious uneven enhancement, the pharyngeal cavity was narrowed to the right, the left submandibular gland was compressed and moved outward, and the left common carotid artery and internal and external carotid artery were compressed and moved outward.

Owing to the lack of specificity in clinical and imaging findings, these modalities are primarily used to determine the extent of surgical resection. On CT, the main manifestations are elliptical or semilunar solid masses with clear boundaries, often accompanied by scattered calcifications (**Figure 2**). On magnetic resonance imaging (MRI), GN mostly presents as low signal intensity on T1-weighted imaging (T1WI) and heterogeneous high signal intensity on T2-weighted imaging (T2WI). Contrast-enhanced scans can show different degrees of delayed enhancement (**Figure 4**) (7). The imaging findings in this patient were typical: cervical CT revealed an oval, slightly low-density shadow with uneven density between the left parapharyngeal space and carotid vascular sheath, and an enhanced scan showed heterogeneous enhancement. MRI of the neck revealed a mass shadow with long T1 and long T2 signals in the left parapharyngeal space, with heterogeneous signal intensity. Multiple patches and other T2 signal shadows could be seen within the mass, and the enhanced scan showed obvious heterogeneous enhancement.

Cervical GN is difficult to distinguish from schwannomas, neurofibromas, paragangliomas, etc., and can be preliminarily distinguished based on clinical manifestations and imaging findings. Schwannomas commonly presents with chronic nerve pain, <c>while</c> ultrasound examination shows that the compressed nerve trunk is tubular hypoechoic, showing the “rat tail sign.” Paragangliomas mostly originate from sympathetic or parasympathetic ganglion cells. MRI showed isointense or low signal intensity on T1WI and isointense or high signal intensity on T2WI. In the present case, the tumor was hard in texture and <c>gray</c> on the cut surface. MRI revealed a low signal on T1W1 and a target sign on T2W1 (external high signal and internal low signal). Meanwhile, GN requires differentiation from neuroblastoma and ganglioneuroblastoma (8). GN is a benign, well-differentiated tumor consisting of mature ganglion cells and abundant Schwann cell stroma, lacking immature neuroblasts. Neuroblastoma is a malignant undifferentiated small round cell tumor composed of immature neuroblasts, with frequent necrosis and sparse stroma. Ganglioneuroblastoma is an intermediate-grade malignant tumor containing both mature and immature neuroblasts. Final diagnosis depends on postoperative histopathology and immunohistochemistry.

**Figure 5:**
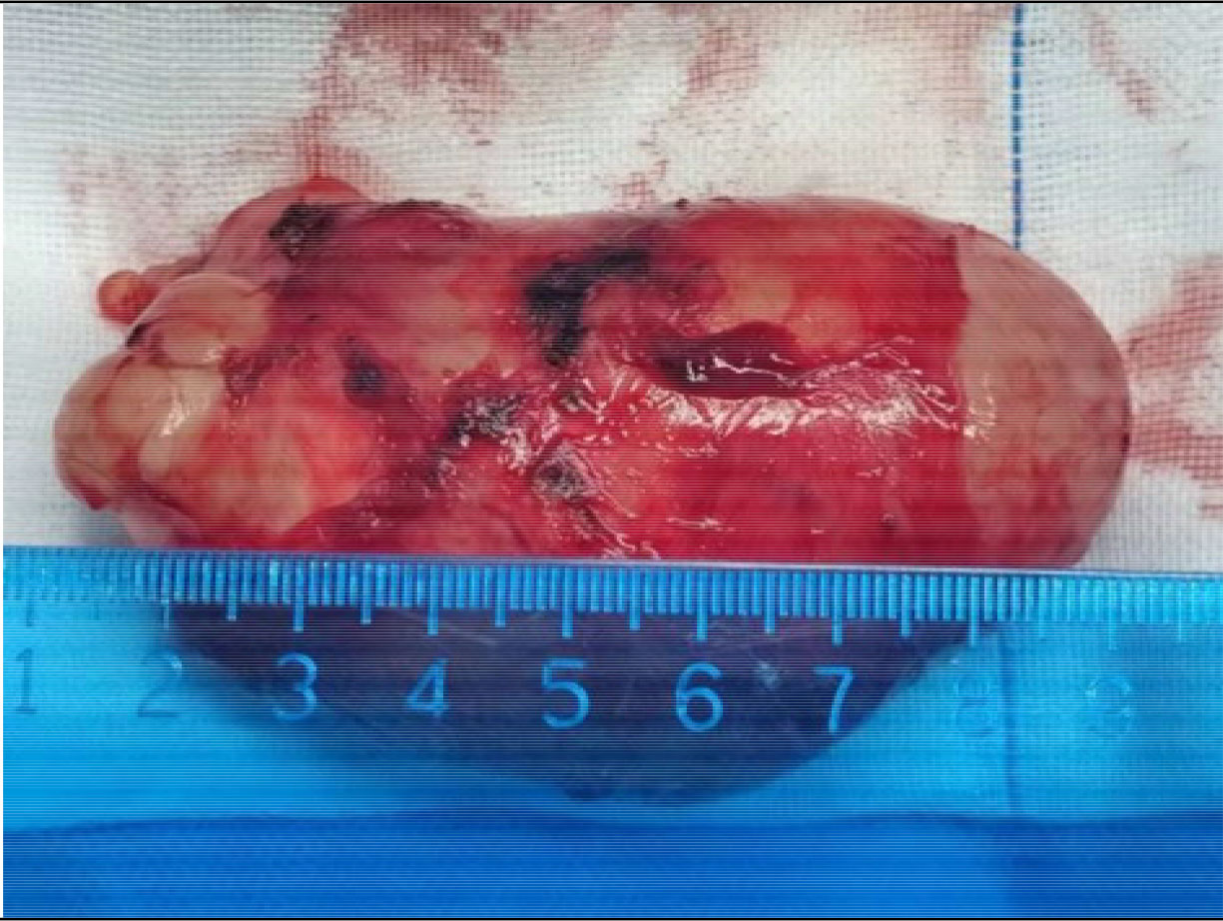
The resected tumor after surgery.

Owing to the rarity of cervical GN and the lack of standardized evidence-based medical support, surgical resection remains the primary treatment (9, 10). Generally, a lateral cervical surgical approach is selected, and a few choose the transoral and transcervical approaches combined with mandibular tilting. The transoral approach is minimally invasive, but is not suitable for large tumors. For large tumors, the transcervical approach, which can remove tumors under direct visualization, is currently the preferred surgical method. However, when the tumor was large and located high, a transcervical approach combined with mandibular flip surgery was selected to fully expose the tumor. In this case, a difficult airway was considered and tracheotomy was performed first. The external cervical approach was selected because the tumor was large and the highest point was located at the level of the soft palate. Curved incisions were made from the upper to the left mastoid, and from the lower to the right thyroid cartilage lamina. During surgery, the tumor was located in the left parapharyngeal space, up to the jugular foramen area of the skull base, and down to the lower edge of the thyroid cartilage, with a complete capsule and no obvious adhesions. The anterior branches of the external carotid artery (superior thyroid, lingual, and facial arteries) were ligated and transected, the posterior bellies of the digastric and stylohyoid muscles were transected, the parapharyngeal space was fully exposed, and the mass was explored. Considering that the mass originated from the cervical sympathetic trunk, the hypoglossal nerve was retracted upward, the cervical sheath and vagus nerve were pulled backwards, and the mass was completely resected. The tumor measured approximately 9.0*5.0*4.0 cm (**Figure 5**).

**Figure 6:**
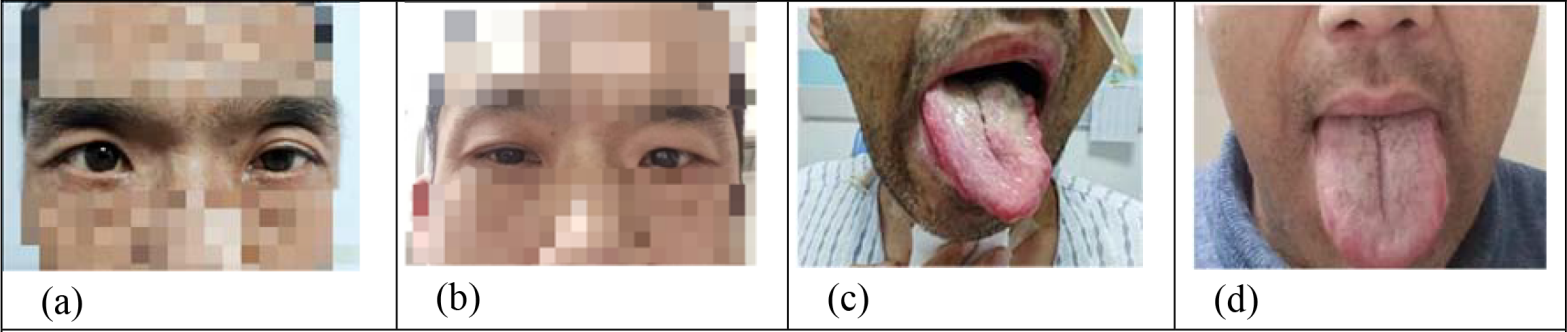
**(a, c)** Photographs of the patient 7 days after surgery. **(b, d)** The patient was followed up for 5 months, and the comparison showed that ptosis and tongue extension were significantly improved.

**Figure 7:**
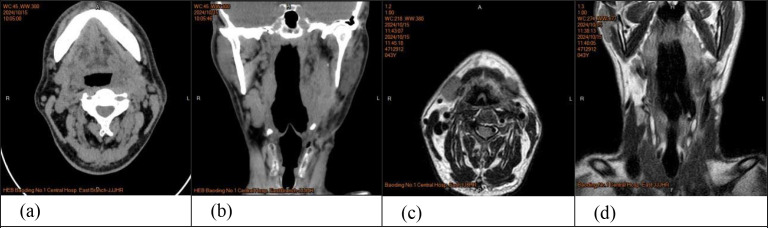
**(a, b)** Neck CT at two months after surgery: The soft tissue of the left lateral wall of the oropharynx was slightly thickened, the structure was less regular, and the surrounding fat space was blurred. The shape of the laryngeal cavity was regular and symmetrical, and the pharyngeal recess, pyriform sinus, and epiglottis valley were present. The epiglottic cartilage, thyroid cartilage and cricoid cartilage were regular in shape. There was no obvious abnormal density shadow in either lobes of the thyroid gland, and the trachea was centered. **(c, d)** Neck MRI at two months after surgery: The local subcutaneous fat space in the left submandibular area was blurred, and the patchy T2 fat-suppression high signal was seen. The rest of the neck structure was still clear, and no obvious abnormal signal was found.

Postoperative recovery of cervical GN is good, and relapse is rare. However, postoperative nerve injury may occur, as lesions predominantly arise from the sympathetic nerve and are located in the upper neck and parapharyngeal skull base area, through which the internal jugular artery and vein, external carotid artery, and cranial nerves IX to XII all pass. These symptoms often disappear spontaneously because the nerves are not completely transected during the operation. In the present case, Horner syndrome, left deviation of tongue protrusion, and dysphagia occurred postoperatively (**Figure 6**), which was considered to be caused by traction injury to the sympathetic, hypoglossal, vagus, and glossopharyngeal nerves during surgery. During the follow-up period, symptoms such as tongue extension to the left and dysphagia almost disappeared, and ptosis was alleviated to some extent (**Figure 6**). Postoperative imaging revealed clear oropharyngeal structures. Cervical CT revealed that the soft tissue of the left lateral wall of the oropharynx was slightly thickened, the structure was irregular, and there was no obvious abnormal density in the remaining structures(**Figure 7**). Cervical MRI showed that the local subcutaneous fat space in the left submandibular region was blurred, there were patches of T2-weighted fat-suppressed imaging, and no obvious abnormal signals were found in other neck structures (**Figure 7**).

## 4 Conclusion

GN is a relatively rare neurogenic tumor. Preoperative diagnosis is challenging and predominantly depends on the histopathological examination of postoperative specimens. Therefore, clinicians should be aware of this differential diagnosis. Surgical resection remains the primary treatment modality, with a favorable prognosis. Postoperative complications should also be considered.

## Data Availability

The original contributions presented in the study are included in the article/supplementary material. Further inquiries can be directed to the corresponding author.
